# A coupled atrioventricular-aortic setup for in-vitro hemodynamic study of the systemic circulation: Design, fabrication, and physiological relevancy

**DOI:** 10.1371/journal.pone.0267765

**Published:** 2022-11-04

**Authors:** Rashid Alavi, Arian Aghilinejad, Heng Wei, Soha Niroumandi, Seth Wieman, Niema M. Pahlevan

**Affiliations:** 1 Department of Aerospace and Mechanical Engineering, University of Southern California, Los Angeles, CA, United States of America; 2 Dornsife/Viterbi Machine Shop, University of Southern California, Los Angeles, CA, United States of America; 3 Division of Cardiovascular Medicine, Keck School of Medicine, University of Southern California, Los Angeles, CA, United States of America; York University, CANADA

## Abstract

In-vitro models of the systemic circulation have gained a lot of interest for fundamental understanding of cardiovascular dynamics and for applied hemodynamic research. In this study, we introduce a physiologically accurate in-vitro hydraulic setup that models the hemodynamics of the coupled atrioventricular-aortic system. This unique experimental simulator has three major components: 1) an arterial system consisting of a human-scale artificial aorta along with the main branches, 2) an artificial left ventricle (LV) sac connected to a programmable piston-in-cylinder pump for simulating cardiac contraction and relaxation, and 3) an artificial left atrium (LA). The setup is designed in such a way that the basal LV is directly connected to the aortic root via an aortic valve, and to the LA via an artificial mitral valve. As a result, two-way hemodynamic couplings can be achieved for studying the effects that the LV, aorta, and LA have on each other. The collected pressure and flow measurements from this setup demonstrate a remarkable correspondence to clinical hemodynamics. We also investigate the physiological relevancies of isolated effects on cardiovascular hemodynamics of various major global parameters found in the circulatory system, including LV contractility, LV preload, heart rate, aortic compliance, and peripheral resistance. Subsequent control over such parameters ultimately captures physiological hemodynamic effects of LV systolic dysfunction, preload (cardiac) diseases, and afterload (arterial) diseases. The detailed design and fabrication of the proposed setup is also provided.

## 1. Introduction

The performance of the human circulatory system cannot be fully comprehended without understanding the interactions of the left ventricle (LV), the left atrium (LA), and the arterial system [1]. The abnormal coupling of the LV-aorta system and the LV-LA system has been shown to contribute to the pathophysiology of system-level cardiovascular diseases [2–6]. In healthy conditions, there exists an optimal hemodynamic balance between the LV, the arterial network (aorta and its vascular branches), and the LA that guarantees delivery of the cardiac output with minimal energy expenditure and modest pulsatility in flow and pressure [2,6–10]. Optimal atrioventricular-aortic hemodynamic coupling can be impaired due to age-related or disease-related changes. For example, recent clinical studies have shown that stiffening of the proximal aorta is associated with pulsatile load on the heart and can lead to the development of heart failure [3,6,11,12]. While the physiological importance of such optimal hemodynamic balance between the LV, the LA, and the vascular network has been shown extensively [2,3,7,13–15], the effects of the individual contributions from ventricular, atrial, and arterial parameters towards generating pressure and flow waves remain unexplored. In order to study the isolated effects of such parameters, it is essential to keep other parameters of the circulation either constant or under control.

In-vitro fluid dynamics studies of cardiovascular systems have shown to be effective in understanding the underlying hemodynamics mechanisms of diseases [4,16–21]. These setups can reduce the high expenses and risks associated with clinical trials, can facilitate the test of diagnostic and therapeutic devices, and can be used to validate computational models of physiological phenomena [22–24]. In addition, such models allow one to study one parameter at a time while controlling all other parameters in a physical setting. This provides a unique advantage over pre-clinical models (e.g., rat models), where complex physiological interactions and biological variabilities exist [17]. Previous studies have introduced various in-vitro hemodynamic simulators and have demonstrated the physiological relevancies of their setups [4,17,25,26]. However, none of these in-vitro simulators account for direct two-way coupling between both the LV-aorta and LV-LA subsystems.

This manuscript introduces a novel in-vitro hydro-mechanical system that is well-suited for studying hemodynamic couplings of the LV-arterial system. This experimental setup is designed to investigate the pulsatile hemodynamics and arterial wave reflections in the (human-scale) systemic circulation. In the setup, the main determinants of cardiovascular function can be controlled by: *i*) the contractile state of the LV; *ii*) the afterload (determined by vascular stiffness and the total peripheral resistance (TPR)); and *iii*) the LV preload (quantified by left ventricular end-diastolic pressure (LVEDP)). In our design, the basal LV is directly connected to the aortic root on the arterial side and to the artificial left atrium (LA) on the atrial side, with aortic and mitral valves in between. In this manuscript, the design and fabrication of the proposed setup are described in detail. The physiological accuracy and relevancy of the experimental setup are also discussed across a wide range of healthy and diseased conditions.

## 2. Materials and method

### 2.1. Description of in-vitro experimental setup

#### Hydraulic circuit components

The main components of our in-vitro experimental setup are *i*) the atrioventricular simulator; *ii*) the aortic simulator; *iii*) the total arterial resistance and compliance simulator; and *iv*) the venous simulator. The atrioventricular simulator consists of a compliant LV sac that is connected to the aortic root on one side via an artificial aortic valve and that is connected to the artificial LA on the other side via an artificial mitral valve (*Medtronic MOSAIC® 305 CINCH®*). The LV sac employed in this simulator is installed inside an LV chamber that connects the artificial LV to a computer-controlled piston-in-cylinder pulsatile pump (*ViVitro* Labs Inc, SuperPump, AR SERIES). The aortic simulator includes the artificial aorta and the end-organ simulators at the terminus of each branch, the latter of which consist of half-filled air syringes and clamps mounted at the outlets in order to account for the compliance and resistance of the eliminated vasculatures. The total arterial resistance and compliance simulator consists of a pair of half-filled air chambers from acrylic glass connected to each other with a resistance valve in between. In order to control the compliance of the system through the compliance chambers, pairs consisting of a pressure bulb (for applying the pressure) and a pressure gauge (for monitoring of the pressure) are mounted on top of each chamber. The height of the air column inside the compliance chambers, as well as the syringes, are adjusted as has been described in previous work [4]. The venous reservior consists of an open tank that is connected to the LA.

#### Hydraulic circuit functioning

**Fig 1:**
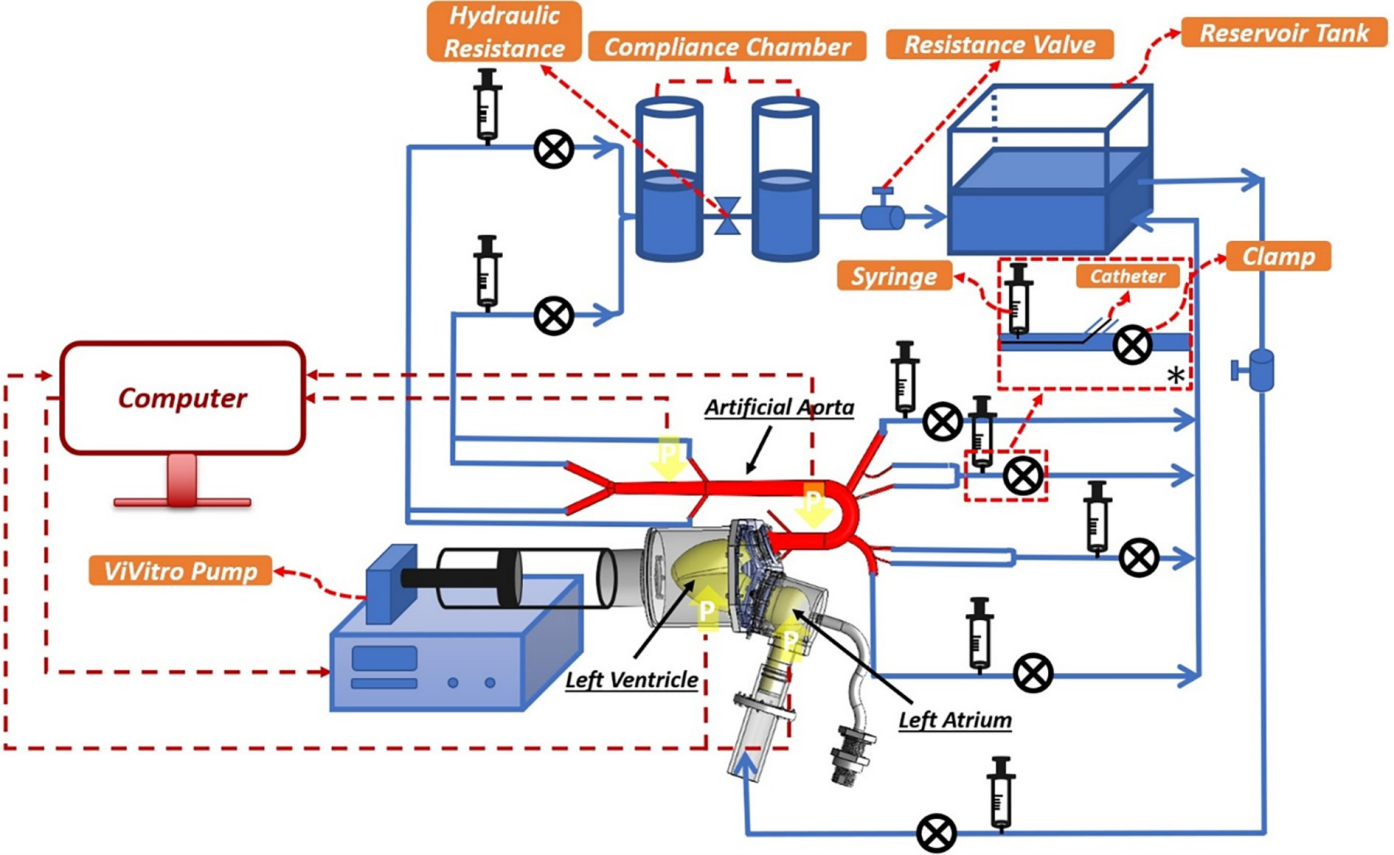
Schematic of the full hydralutic circuit corresponding to the left atrioventricular-aortic hemodynamic simulator setup and its circulation path. The schematic includes the complete in-vitro circulation system consisting of the LV, the aorta, the vascular components of the systemic circulation, and the left atrium (*: Schematic of a sample outlet unit).

The compliant LV sac is contracted (squeezed) inside a plexiglass container using a pulsatile pump programmed by the ViVigen interface (*ViVitro* Labs Inc.). Using physiological input waveforms for the piston pump displacement (as shown in S1 File), the fluid outside of the LV sac is pressurized (LV contraction) and depressurized (LV relaxation) as the piston moves. After the pressure starts to rise inside the LV sac, the prosthetic aortic valve opens, and the test fluid enters the aorta from the LV and begins to generate hemodynamic waves that propagate down the aorta. The fluid is then exited from the aorta and its branches via the two compliance chambers that are installed at the end of the aortic loop. The testing fluid then flows from the two compliance chambers into the reservoir tank, which then flows towards the artificial LA. As the LV starts to relax (due to the depressurization inside the LV chamber), the mechanical mitral valve opens, and the fluid subsequently pumps back inside the LV. This completes the in-vitro cardiac loop. A schematic of the full hydralutic circuit corresponding to the left atrioventricular-aortic hemodynamic simulator setup and its corresponding circulation path is presented in Fig 1.

#### Design considerations for LV-aorta and LV-LA hemodynamic couplings

**Fig 2:**
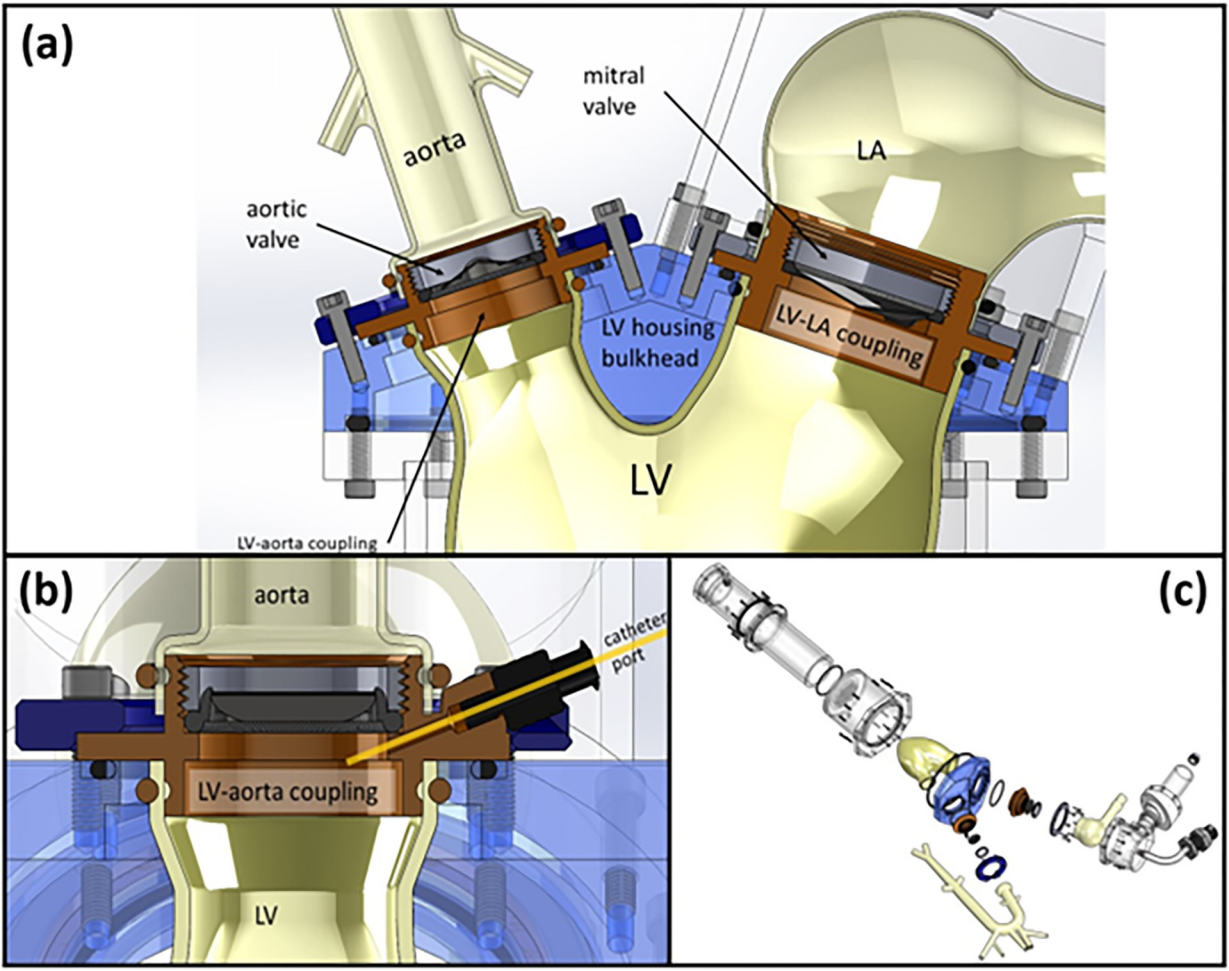
Detailed schematics of the LV-LA and the LV-aorta couplings. **(a)** Acetal plastic inserts placed in the LV housing bulkhead serve as both LV-LA and LV-aorta couplings as well as housings for their respective mitral and aortic valves. **(b)** A cross-sectional view of the LV-aorta coupling illustrates how the LV-aorta coupling insert includes a duct through which a catheter may be inserted for LV internal pressure measurements. **(c)** Exploded view of the setup components corresponding to the LV-aorta and LV-LA coupling mechanisms.

In the design process, we have ensured that the two-way couplings between the LV, LA, and aorta are captured by the in-vitro setup. As such, for the LV-aorta interface design, the basal LV is directly coupled with the aortic root through a prosthetic aortic valve. The LV-aortic coupling is machined from rigid acetal plastic that also serves as housing for the aortic valve (Fig 2A). In the LV-LA interface design, the artificial LV is coupled with the artificial LA on the atrial side through a mechanical mitral valve. The LV-LA coupling is also fabricated from rigid acetal plastic and serves as housing for the mitral valve (Fig 2A). Each of the coupling pieces mount to a bulkhead on the LV-housing with an O-ring sealed flange. Grooved sleeves extending from either side of the coupling flanges provide sealing surfaces for the compliant cuffs of the LV, LA, and aorta. O-rings that are placed under tension around the cuffs at the grooved portion of the sleeves complete this seal. The mechanical or prosthetic valves are sealed into the couplings by compressing their suture rings against inner rims on the couplings with a threaded retaining ring. The LV-aorta coupling includes a Luer-lock compatible port (Fig 2B) through which a catheter can be inserted for measuring LV internal pressure. Efforts were made to keep the couplings as short as possible in order to minimize usage of rigid material along the LA-LV-aorta flow path. This coupling/valve housing approach is easily adapted to different LV, LA, and aorta sizes as well as a variety of mechanical or prosthetic valve types. Overall, the two-way couplings are mainly provided via the artificial valves and direct atrioventricular-aortic couplings with minimal inter-connectors and a cohesive media.

**Fig 3:**
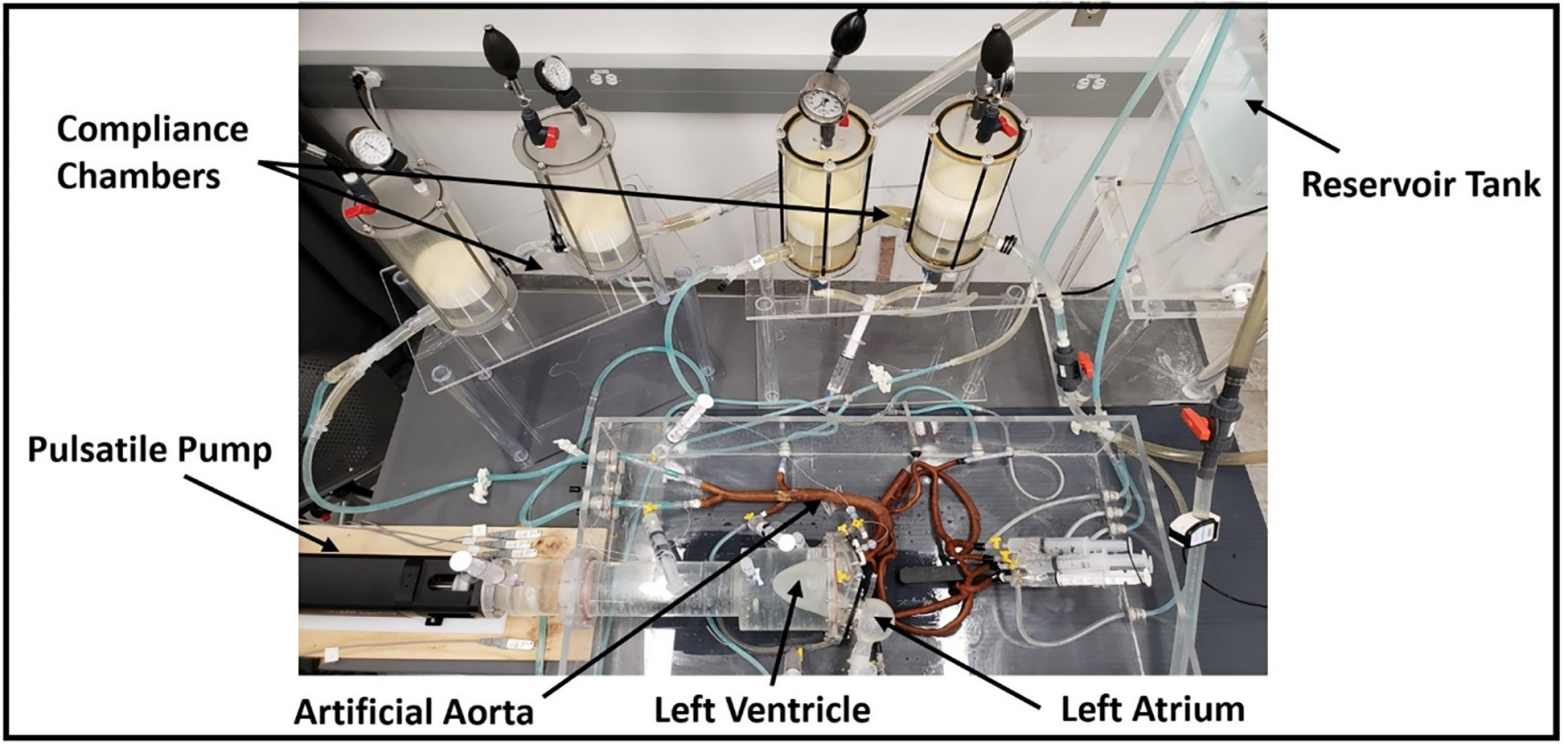
Picture of the complete in-vitro circulation system consisting of the LV, the aorta, the vascular components of the systemic circulation, and the left atrium.

A picture of the final fabricated setup is shown in Fig 3. The detailed CAD design of the constructed setup can be found in the manuscript’s Supplementary Material (see S2 File).

### 2.2. Artificial organ fabrication

**Fig 4:**
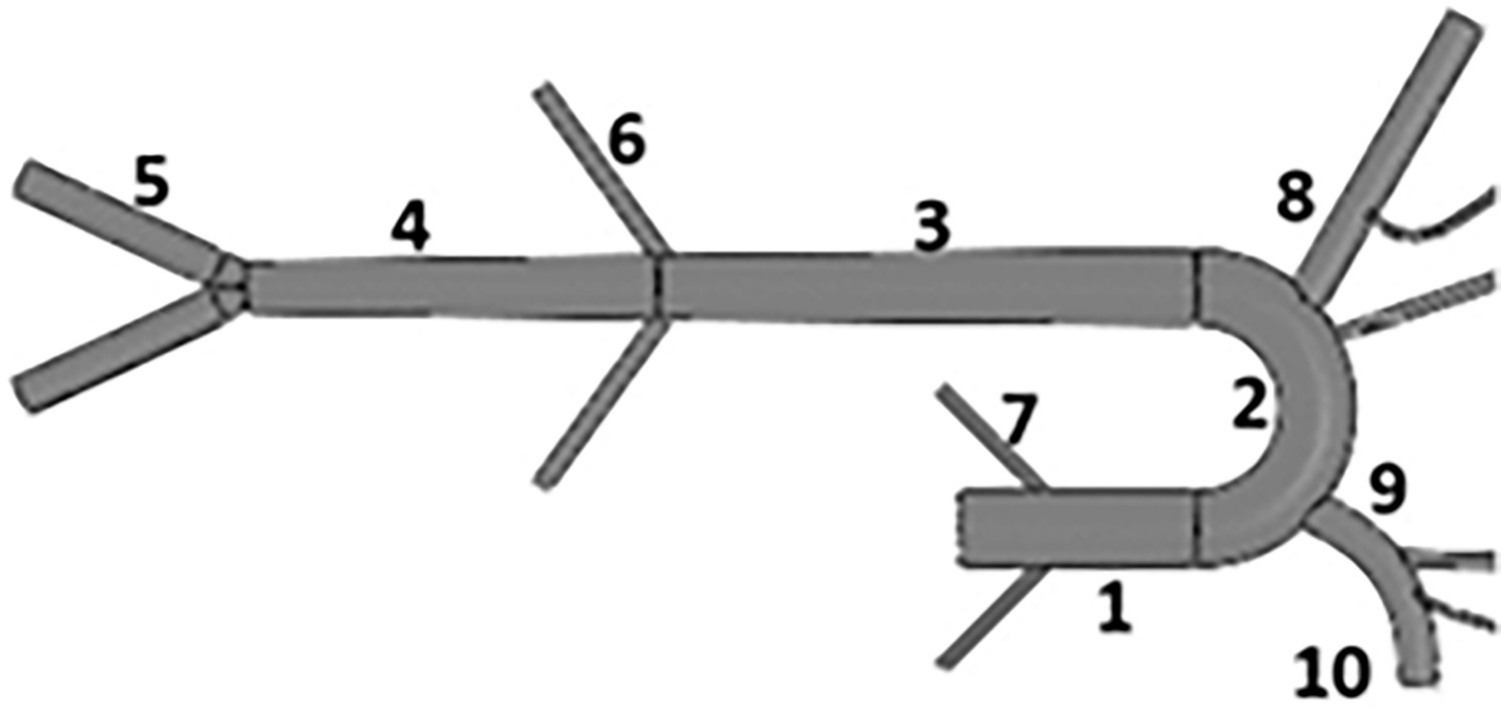
Schematic and segment diagram of the aorta mold.

The artificial organs installed in the setup (e.g., aorta and LA) are fabricated using natural latex rubber (*Chemionics Corp*.) and silicone rubber (RTV-3040, *Freeman Manufacturing & Supply Company*). These materials are chosen based on their characteristic ability to replicate the stiffness of a physiological aorta [4]. The artificial aortas are fabricated in-house based on the one-to-one human-scale molds consisting of the ascending aorta, the aortic arch, the thoracic aorta, the abdominal aorta, and all the major branches (e.g., coronary and renal arteries). The aortic molds are built using either polyvinyl alcohol (PVA) via a 3D printer (*Ultimaker S5 Dual Extrusion*) or using a stainless-steel metal mold. Fig 4 and Table 1 list the segments and corresponding dimensions of the aortic molds that are employed in this work.

**Table 1:** Geometric characteristics of the aorta mold.

Number	Segment Name	Length (mm)	Inlet Diameter (mm)
1	Ascending Aorta	70	24.0
2	Aortic Arch	60	18.0
3	Thoracic Aorta	170	20.0
4	Abdominal Aorta	130	16.0
5	Iliac Artery	76	12.0
6	Renal Artery	65	5.0
7	Coronary Artery	45	4.0
8	Left Subclavian Artery	100	8.4
9	Brachiocephalic Artery	34	12.4
10	Right Subclavian Artery	65	8.4

Dipping and coating casting methods are used for the natural latex and silicone-based fabrications, respectively. For the latex-based aortas, the following fabrication steps are taken: *i*) dipping the aortic mold inside the liquid latex container; *ii*) removing the coated mold from the container; *iii*) curing the coated material for 2 hours in standard room temperature (25°C); *iv*) repeating the coating for more layers (as necessary) so as to achieve the desired aortic compliance (steps *i* to *iii*). For the silicone-based fabrications, the following steps are taken: *i*) mixing the catalyst and the base of the silicon rubber (RTV-3040, *Freeman Manufacturing & Supply Company*) with a mass ratio of 10 (base) to 1 (catalyst); *ii*) removing the mixture bubbles using a vacuum pump chamber; *iii*) coating a light silicon sheet using 25 g of the mixed solution using a soft-tip acrylic brush; *iv*) curing the coated material for 16 hours at standard room temperature (25℃); *v*) repeating the coating for more layers (as necessary) so as to achieve the desired aortic compliance (steps *i* to *iv*). For ensuring surface uniformity, the mold is turned upside down at each drying step. Table 2 presents the measured pulse wave velocities (PWV) and aortic compliances (AC) of the final fabricated aortas employing either silicone or latex. AC is measured by adding incremental volumes of fluid and measuring the corresponding incremental change in pressure. Details on acquiring PWV measurements are described in Section 2.3.

**Table 2:** Dynamic and physiological properties of the fabricated aortas.

Aorta No.	PWV (m/s)	AC (mL/mmHg)	Material
Aorta-1	5.6	2.28	Silicone
Aorta-2	10.6	1.78	Latex
Aorta-3	12.6	1.51	Latex
Aorta-4	16.6	1.43	Latex
Aorta-5	17.3	1.31	Latex
Aorta-6	18.0	1.19	Latex
Aorta-7	19.7	0.83	Latex
Aorta-8	21.7	0.71	Silicone

After casting the models using either latex or silicone, the prepared aortas are removed from their respective molds. For the metal molds, removal is achieved through gentle injection of water at the material/mold interface. For PVA molds, removal is achieved by immersing the models inside water at standard room temperature for 96 hours. The PVA mold is then dissolved in the water, and the PVA residue is manually detached from the latex or silicon aorta at the end of the process. Lastly, to enhance surface quality, the latex aortas are submerged in Clorox bleach for 12 hours, followed by another 12 hours of submerging in water for the final curing process.

The materials and molds described above are chosen based on their ability to provide physiological results as well as adding flexibility for further studies on cardiovascular fluid dynamics. Both materials (silicone and latex) provide characteristics with the ability to replicate compliance and PWVs of physiological aortas as shown in Table 2. Silicone enables the production of transparent aortas which are preferable for optical methods for flow visualization such as particle image velocimetry. On the other hand, latex provides more control over the compliance and PWV of fabricated aortas. There is no difference in the final product as one uses either metal or PVA molds. Metal mold has previously shown to provide reproducible and reliable models for producing simplified artificial aortas, while PVA molds can be potentially used for patient-specific fabrications of the aorta [4,13,27].

Fabrication procedures for the LV and LA are similar to the fabrication procedure of the silicone-based aortas. The designed molds are printed for each of LV and LA via a 3D printer using PVA material, and the same steps as the aforementioned fabrication steps for the silicone-based aortas are taken. For fabricating artificial organs (i.e., aortas, LV and LA) in this work, elastomer-based materials, i.e., silicone or natural latex, have been used. Elastomers are rubber-like materials which are categorized as soft materials with a low Young’s modulus, so the fabricated artificial organs are able to return to their initial condition when the external stress is diminished [28–31]. The control volume of our fabricated LV and LA are 280 mL and 120 mL, respectively.

### 2.3. Procedures and measurements

The in-vitro atrioventricular-aortic simulator has been tested across a wide range of physiological hemodynamic conditions (e.g., heart rate (HR) = 50 to 125 bpm, cardiac output (CO) = 2 to 5 L/min, LVEDP ≈ 0 to 33 mmHg, aortic systolic blood pressure (SBP) ≈ 80 to 170 mmHg, aortic diastolic blood pressure (DBP) ≈ 55 to 90 mmHg) [32–39]. In order to account for the changes in LVEDP, the venous return pressure is increased by adjusting the height of the venous reservoir tank connected to the LA. For assigning cardiac parameters to the setup, user-defined input waveforms are imported into the pulsatile pump controller (ViViGen interface). The frequency of the operation for the pump (which determines the HR) is also modified using the Vivigen interface on the computer unit. The input profile of the pulsatile pump system is adjusted to simulate the impact of LV contractility on system hemodynamics. The simulated contractility ranges from LV-*dp/dt*_*Max*_ = 937.4 mmHg/s to LV-*dp/dt*_*Max*_ = 2558.3 mmHg/s, covering low to high contractility conditions. Using resistance clamps connected to the end-organ outlets, the cross-sectional area of the outlet units is controlled to change the TPR of our atrioventricular-aortic simulator. In order to evaluate the physiological relevancy of the atrioventricular-aortic setup under normal condition, we simulate a baseline of *HR* = 75 bpm, *CO* = 5 L/min, LVEDP = 7 mmHg, SBP = 121.20 mmHg, DBP = 80.78 mmHg, AC = 1.51 mL/mmHg, and *TPR* = 18.85 mmHg∙min/L.

For modeling LV systolic dysfunction, we run the setup under reduced CO conditions. This is achieved by reducing the stroke volume of the LV through adjusting the displacement amplitude of the LV-excitation pump. In order to simulate age-related or disease-related alterations in aortic stiffness, different physiological stiffnesses are achieved by changing the number of applications of dipping (for latex aortas) or coating (for silicone aortas). The corresponding aortic stiffness is quantified by measuring the PWV (the speed at which hemodynamic waves propagate inside the vasculature). Physiologically speaking, PWV depends on the structural alterations (which can be induced by aging) and transient functional changes in the arterial wall [40]. To compute the PWV shown in Table 2, the foot-to-foot method is applied for each one of the aortas using the measured pressures inside the setup. In such a method, the delay time is measured from the foot of the first propagating wave at the aortic root to the foot of the second propagating wave at the aortic bifurcation. The distance between the two sites is a known quantity that is based on the corresponding design of the aortic models.

A list of the parameters that are measured in this study, as well as the devices that have been used for the process of measurement, include: *i*) pressure (via a Millar MIKRO-TIP® Catheter Transducer (*Mikro-Cath*) using a PowerLab 4/35 from ADInstruments); *ii*) flow (via a Transonic Flowmeter (TS410)); and *iii*) heart sound (via a non-invasive wireless optical tonometer called Vivio [41]). In order to assess the effects of each input variable (e.g., LV contractility) on the local and global cardiovascular performance, four measurement sites are selected for the hemodynamic data collection: the central LV, the ascending aorta (6 cm away from the aortic root), the abdominal aorta (23 cm away from the aortic arch center), and the central LA. Water is used as the circulating fluid in all experiments, and any visible air bubbles are removed prior to running experiments. Table 3 lists the range of parameters employed to simulate realistic hemodynamic conditions in our in-vitro simulations.

**Table 3:** Summary of the hemodynamic conditions in the in-vitro experimentations.

Hemodynamic Parameter	Range	Unit
Heart Rate (HR)	[50, 125]	bpm
Cardiac Output (CO)	[2, 5]	L/min
Stroke Volume (SV)	[16, 100]	mL/beat
LV End Diastolic Pressure (LVEDP)	[0, 33]	mmHg
LV Contractility (*LV-dp/dt*_*Max*_)	[937, 2558]	mmHg/s
Aortic Systolic Blood Pressure (SBP)	[80, 170]	mmHg
Aortic Diastolic Blood Pressure (DBP)	[55, 90]	mmHg

### 2.4. Hemodynamic analysis

In this work, we employ both aortic input impedance as well as wave intensity (WI) as metrics to examine the dynamical and physiological accuracy of our proposed experimental setup.

#### Aortic input impedance

Aortic input impedance in pulsatile flow is defined as the ratio of pressure to flow in the frequency domain. It is a measure of the amount by which the aorta ‘impedes’ the flow. Input impedance is an index of arterial properties and is given by the expression [37,40]:

$$z_{in}(\omega )=\frac{p(\omega )}{q(\omega )},$$ (1)

where *ω* is the frequency, *z*_*in*_(*ω*) is input impedance, *p*(*ω*) and *q*(*ω*) are the Fourier representations of the measured pressure and flow computed through applications of the fast Fourier transform (using *fft* in Matlab (Mathworks inc)).

#### Wave intensity

WI analysis is a well-established pulse wave analysis technique for quantifying the energy carried by arterial waves [42]. WI is determined by incremental changes in pressure and flow velocity, and hence requires measurements of both. A typical pattern of WI consists of a large amplitude forward (positive) peak (corresponding to the initial compression caused by a left ventricular contraction) followed by a small amplitude backward (negative) peak (corresponding to reflections from the initial contraction) which itself may be followed by a moderate amplitude forward decompression wave [42,43]. The WI, called *dI*(*t*), is defined by the expression:

dI(t)=dP(t)∙dU(t), (2)

where *t* is the time, *P*(*t*) is the measured pressure, and *U*(*t*) is the measured velocity. For acquiring simultaneous measurements of pressure and flow for quantifying both impedance and WI, we use a Millar pressure system and Transonic flow meter as described in Section 2.3.

## 3. Results

### 3.1. Physiological accuracy of the atrioventricular-aortic hemodynamics

**Fig 5:**
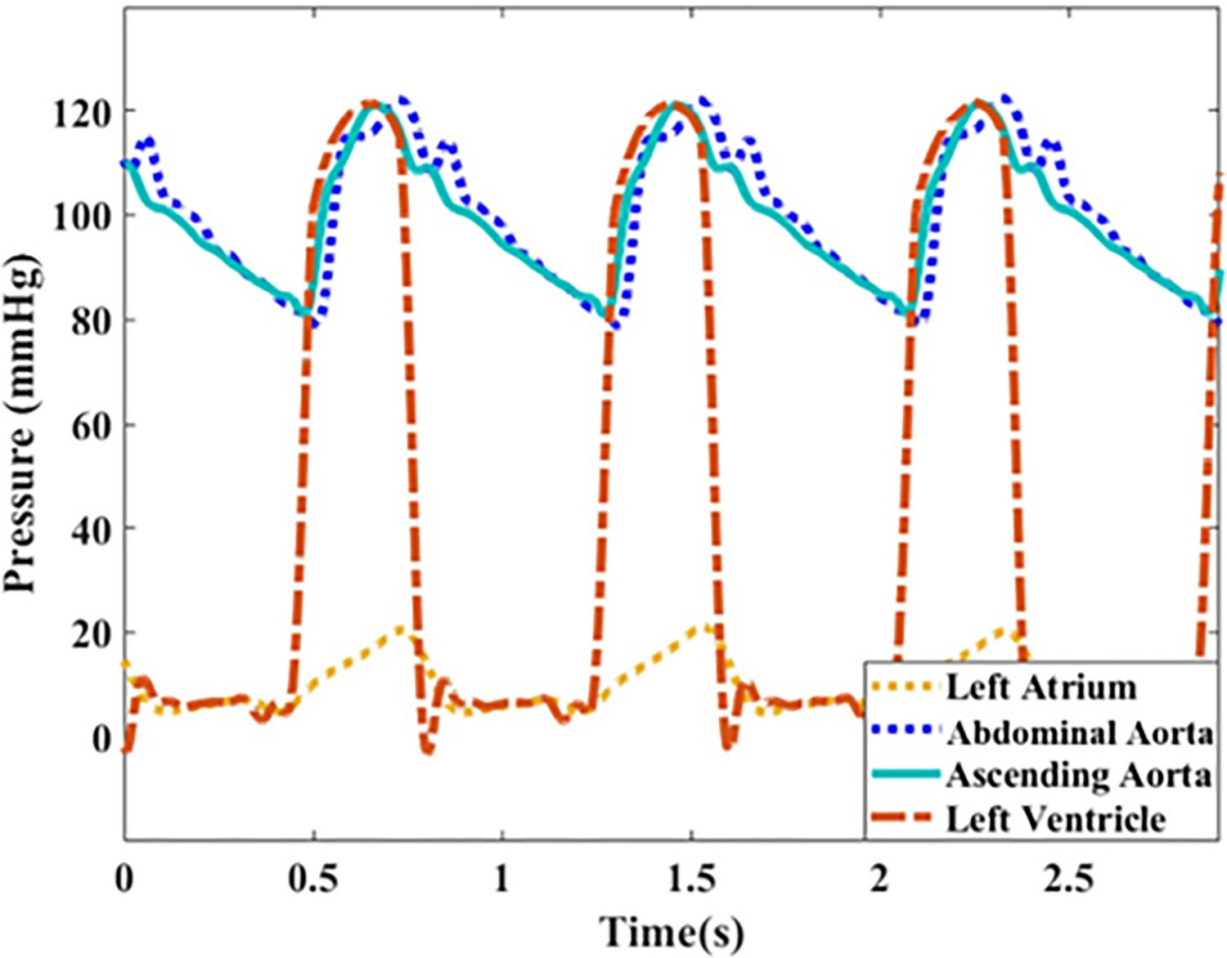
Pressure waveforms at different anatomical sites including the left atrium, the left ventricle, the ascending aorta, and the abdominal aorta. Waveforms are plotted for a normal healthy case (Aorta-3 of Table 2) corresponding to *CO* = 5 L/min and *HR* = 75 bpm (SV = 67 ml/beat).

Fig 5 presents pressure waveforms at the LA, the LV, the ascending aorta, and the abdominal aorta corresponding to a baseline case of a healthy patient (i.e., *HR* = 75 bpm and *CO* = 5 L/min). A mid-range aorta (Aorta-3 of Table 2) is used for this measurement. The baseline LVEDP is 7 mmHg and the computed TPR is 19.32 mmHg.min/L, both of which are consistent with a normal healthy patient [37–40].

**Fig 6:**
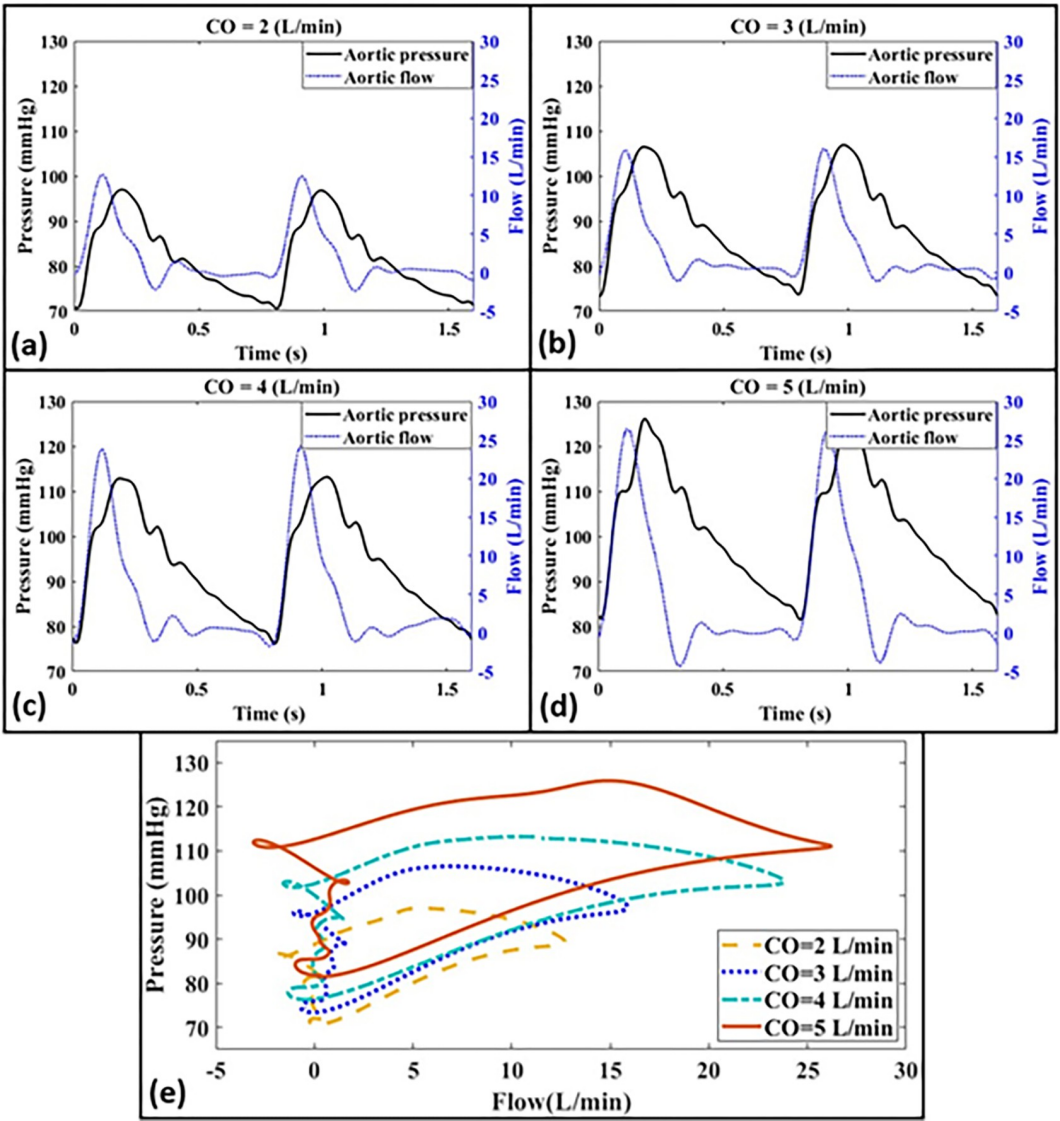
Demonstration of simultaneous aortic pressure and aortic flow measurements for Aorta-3 at different cardiac outputs corresponding to **(a)** *CO* = 2 L/min, **(b)** *CO* = 3 L/min, **(c)** *CO* = 4 L/min, and **(d)** *CO* = 5 L/min. The corresponding pressure-flow loops are also presented in the box **(e)**. Heart rate is 75 bpm for all cases.

Fig 6 presents simultaneous pressure and flow measurements at the ascending aorta for different cardiac outputs (i.e., *CO* = 2, 3, 4, 5 L/min) as well as the corresponding pressure-flow loops (Fig 6E). For these measurements, Aorta-3 and HR = 75 bpm are again employed. The expected fiducial features of the pressure and flow waveforms, including the pressure dicrotic notch (associated with the closure of the aortic valve), pressure augmentation (indicating the inflection point where the backward wave starts superimposing onto the forward wave), and the valve closure effect (nearly zero flow during the diastole), can all be observed in Fig 6. Results demonstrate that our setup is indeed sensitive to the increase in both aortic pressure and flow that arises from an increase in total cardiac output.

**Fig 7:**
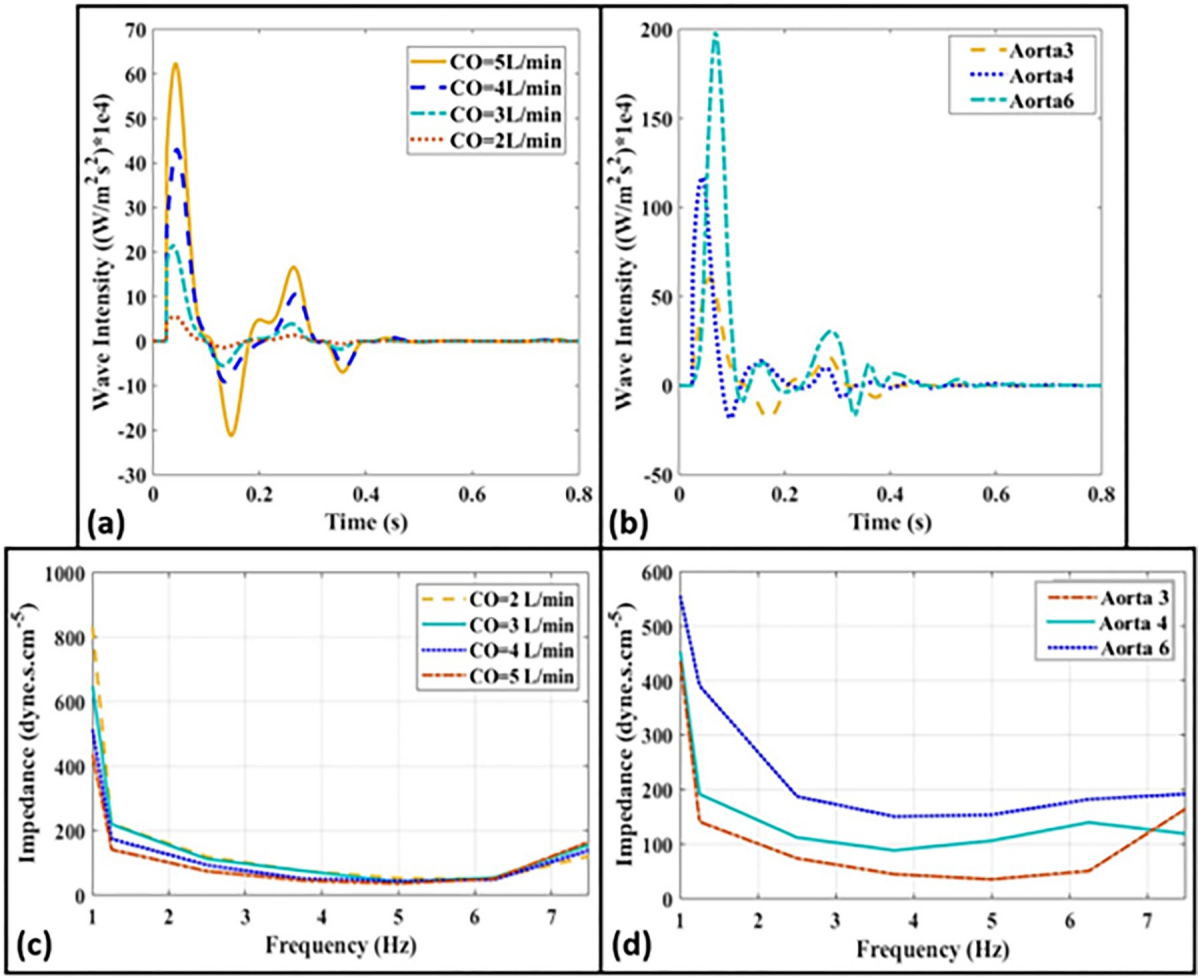
Wave intensity profiles measured at the ascending aorta for **(a)** different cardiac outputs of the same Aorta-3 (*CO* = 2, 3, 4, 5 L/min), **(b)** different aortas for the same *CO* = 5 L/min (Aorta-3, 4, 6). **(c, d)** Aortic input impedances corresponding to the same configurations of **(a,b**). Heart rate is 75 bpm for all the cases.

Fig 7A and 7B provide the corresponding WI (Eq (2)) from the simultaneously-measured pressure and flow waveforms for different cardiac outputs and aortas, respectively. Typically-expected physiological patterns of WI are well-established in literature for clinical data [42]. Such patterns can be used as reference for assessing the physiological correctness of the WI profiles generated by our in-vitro setup and, indeed, our results appear consistent. Fig 7C and 7D additionally present the aortic input impedance for different cardiac outputs of a fixed aorta (Aorta-3) and for different aortas at the same cardiac output (CO = 5 L/min), respectively. All measured data correspond to HR = 75 bpm. The raw data corresponding to all the figures of this section is available online.

### 3.2. Effects of cardiac function on coupled atrioventricular-aortic hemodynamics

**Fig 8:**
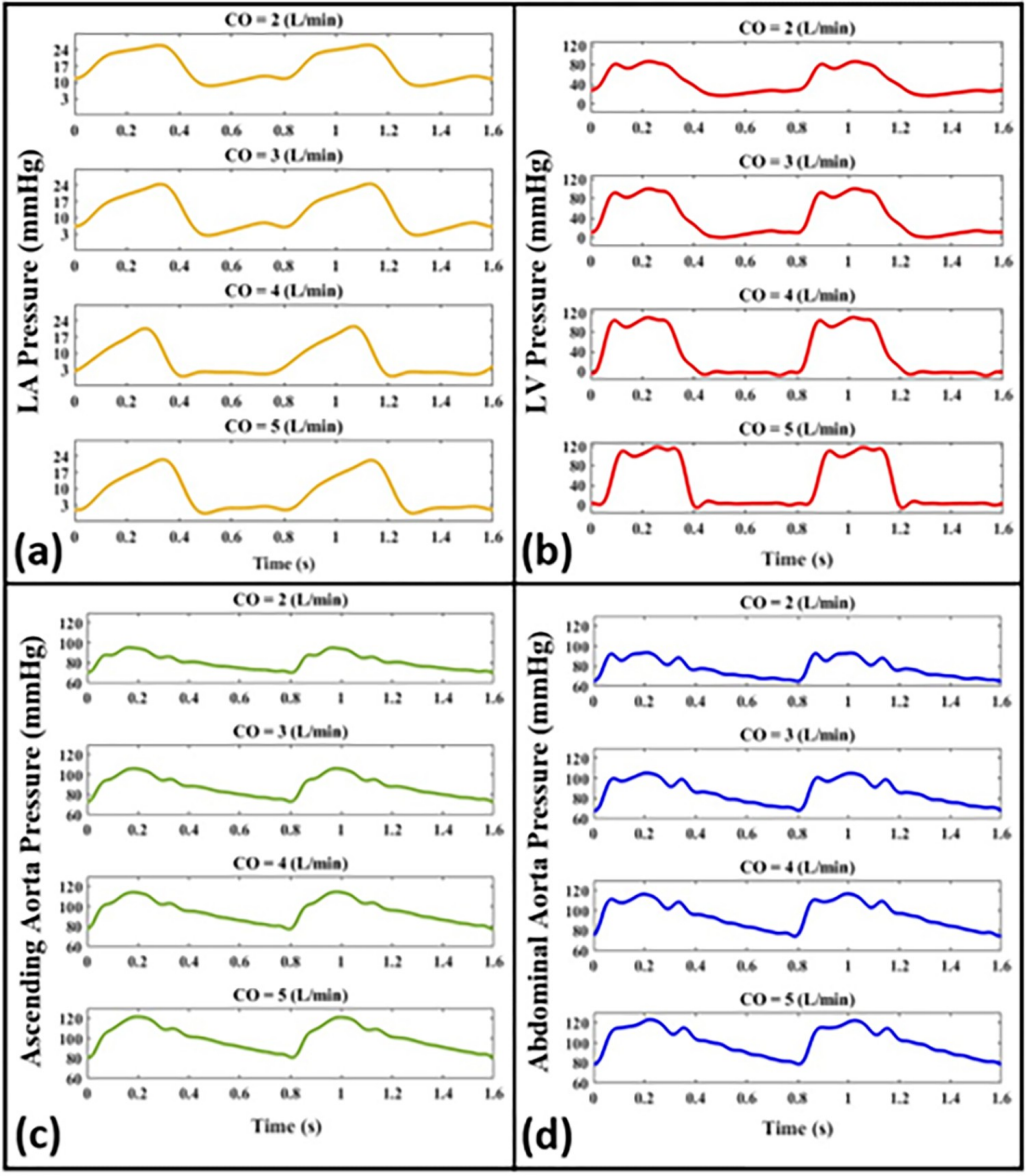
Pressure waveforms for normal (*CO* = 5 L/min) and impaired (*CO* = 2, 3, 4 L/min) conditions of cardiac function at different anatomical sites including **(a)** the left atrium, **(b)** the left ventricle, **(c)** the ascending aorta, and **(d)** the abdominal aorta. Waveforms are plotted for Aorta-3 at *HR* = 75 bpm and a fixed (normal) contractility.

In this section, we study the effects of cardiac function determinants (i.e., stroke volume, contractility, and heart rate) on measured pressure waveforms. Fig 8 presents such measured pressures at different anatomical sites for different stroke volumes corresponding to a normal cardiac output (*CO* = 5 L/min) and reduced cardiac outputs for simulating impaired cardiac function (i.e., *CO* = 2, 3, 4 L/min). Measurements are conducted for Aorta-3 at a fixed (normal) contractility and HR = 75 bpm.

**Fig 9:**
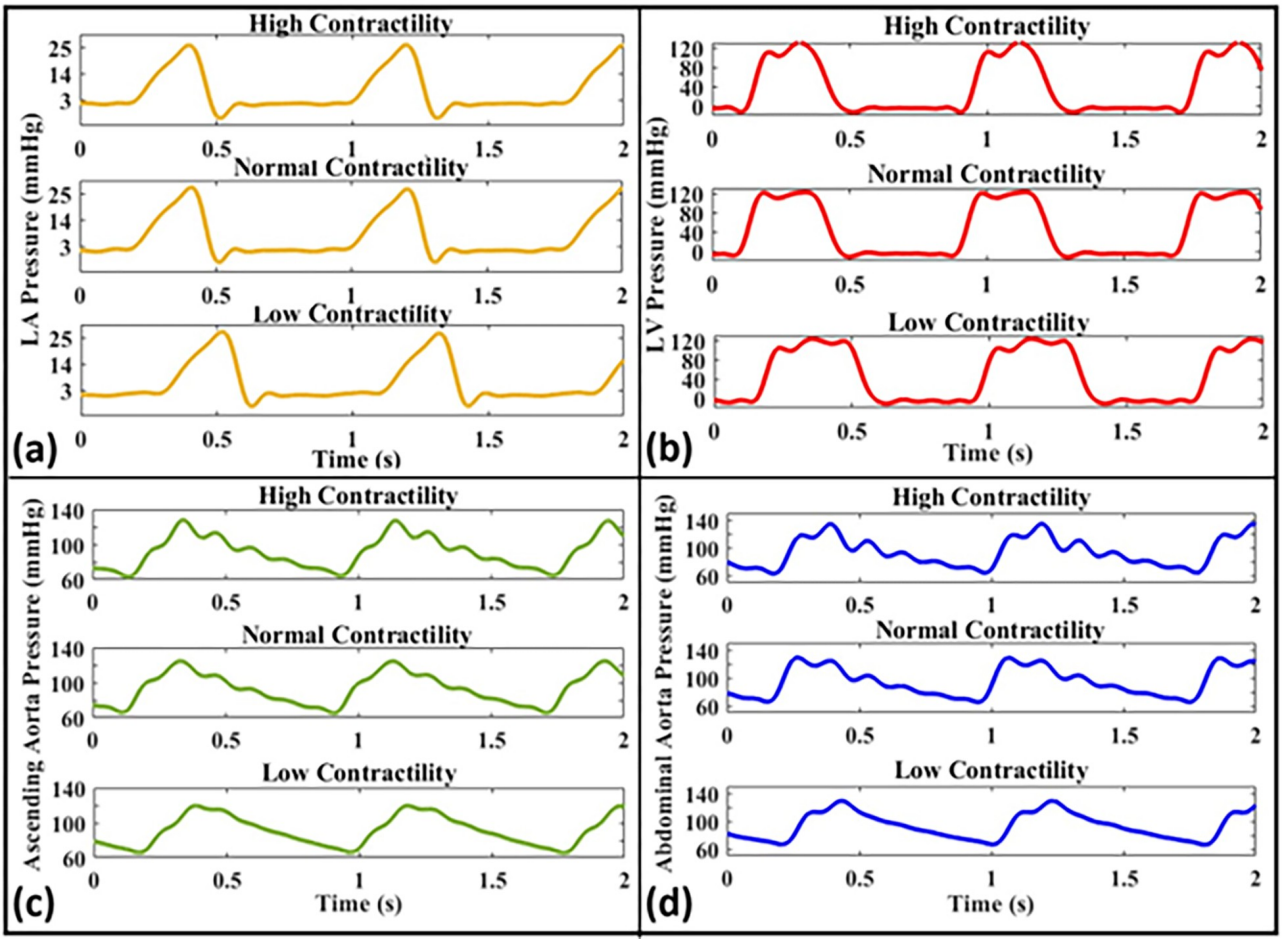
Pressure waveforms for different contractility conditions including high (LV*-dp/dt*_*Max*_ = 2558.3 mmHg/s), normal (LV*-dp/dt*_*Max*_ = 1432.7 mmHg/s), and low contractility (LV*-dp/dt*_*Max*_ = 937.4 mmHg/s) at **(a)** the left atrium, **(b)** the left ventricle, **(c)** the ascending aorta, and **(d)** the abdominal aorta (Aorta-4, HR = 75 bpm, CO = 5 L/min).

Fig 9 presents measured pressure data at the LA, the LV, the ascending aorta, and the abdominal aorta for different contractility conditions, i.e., high contractility (LV-*dp/dt*_*Max*_ = 2558.3 mmHg/s), normal contractility (*LV-dp/dt*_*Max* =_ 1432.7 mmHg/s), and low contractility (*LV-dp/dt*_*Max*_ = 937.4 mmHg/s) at a fixed HR of 75 bpm and CO of 5 L/min.

**Fig 10:**
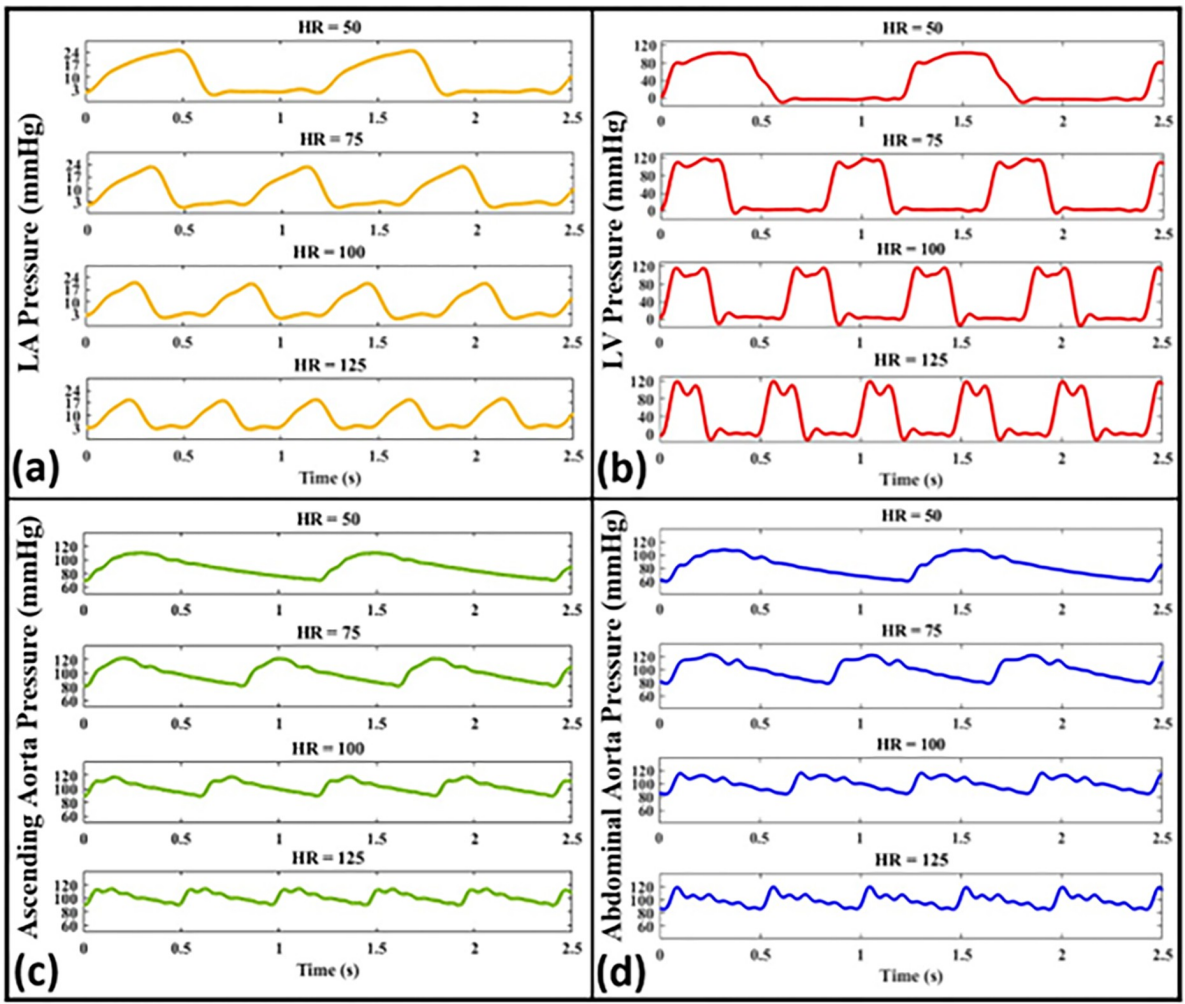
Pressure waveforms for different heart rates (*HR* = 50, 75, 100, 125 bpm) at different anatomical sites including **(a)** the left atrium, **(b)** the left ventricle, **(c)** the ascending aorta, and **(d)** the abdominal aorta (Aorta-3, *CO* = 5 L/min).

The measured pressure waveforms for different heart rates (i.e., *HR* = 50, 75, 100, 125 bpm) at different anatomical sites are presented in Fig 10 for a fixed (normal) contractility condition. These waveforms are measured using Aorta-3 at *CO* = 5 L/min. The raw data corresponding to all figures of this section is available online.

### 3.3. Effects of afterload on coupled atrioventricular-aortic hemodynamics

**Fig 11:**
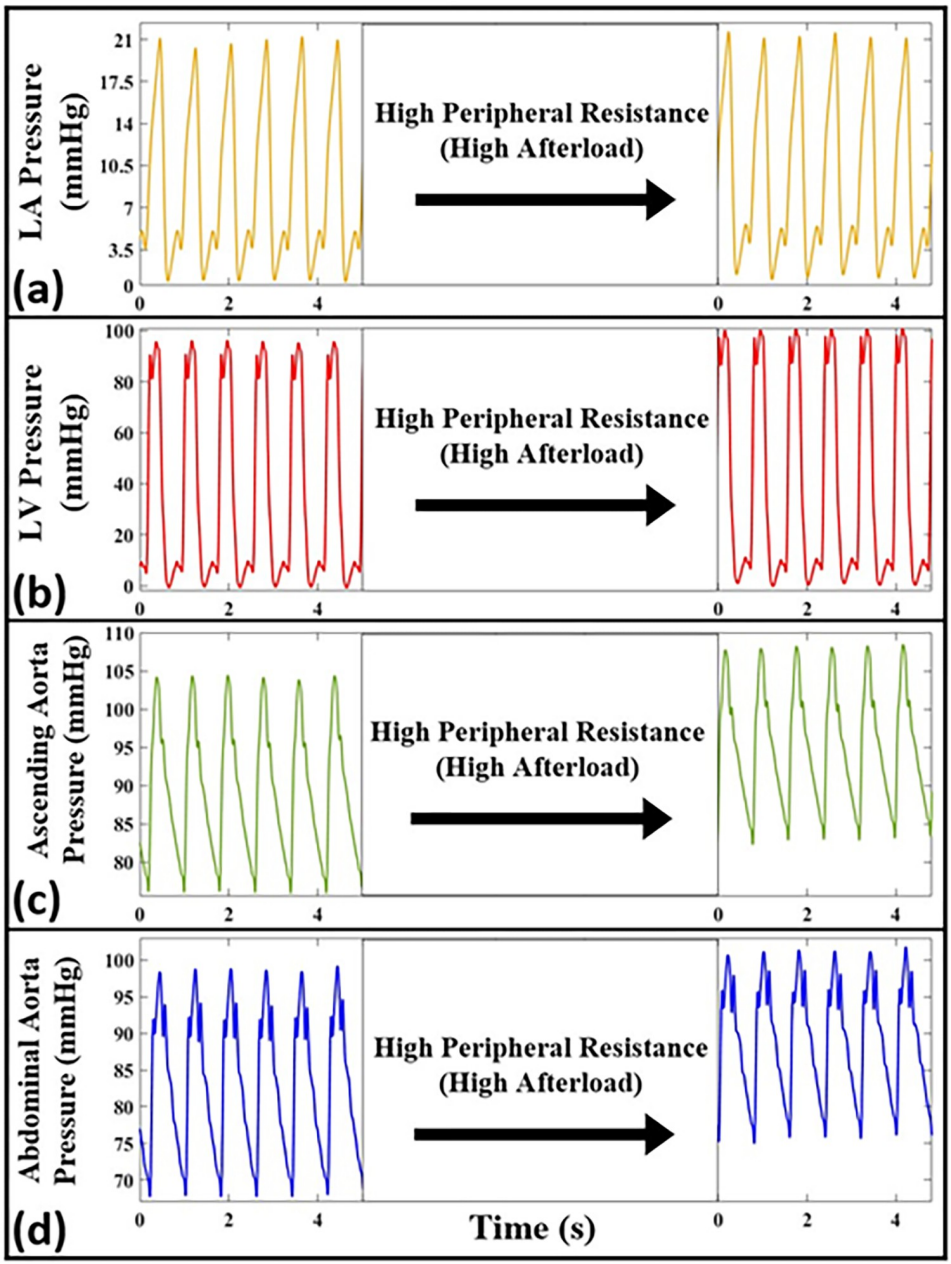
The measured pressure waveforms for normal and high peripheral resistance (i.e., high afterload) conditions at different anatomical sites including (a) the left atrium, (b) the left ventricle, (c) the ascending aorta, and (d) the abdominal aorta. Waveforms are produced from Aorta-3 and correspond to *HR* = 75 bpm. The data of this figure is collected without pausing the pump to show the immediate dynamic changes of the setup (we only showed the oscillatory steady state of each phase for comparison).

In this section, we study using our setup the effects on hemodynamic waves of peripheral resistance and aortic compliance (determinants of the afterload). Fig 11 demonstrates the effect of high peripheral resistance on the measured pressure waveforms inside the LA, the LV, the ascending aorta, and the abdominal aorta. This effect is modeled using the terminal clamps mounted at the outlets of the artificial aorta (as described in Section 2). All data is from Aorta-3 and is simulated at HR = 75 bpm.

**Fig 12:**
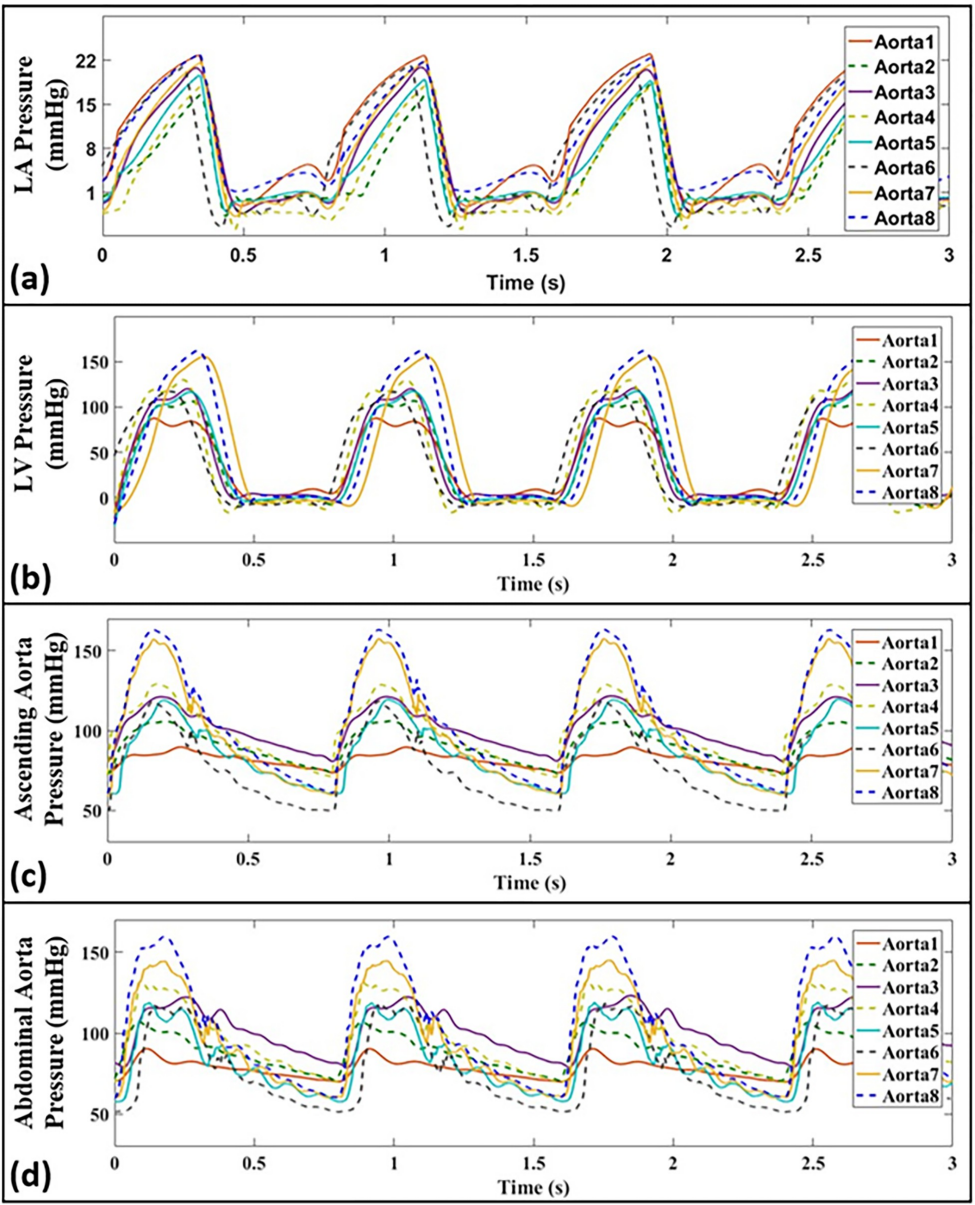
Pressure waveforms, corresponding to different aortas (i.e., Aorta-1 to 8) representing various aortic compliances, at different anatomical sites including **(a)** the left atrium, **(b)** the left ventricle, **(c)** the ascending aorta, and **(d)** the abdominal aorta. Waveforms correspond to *CO* = 5 L/min and *HR* = 75 bpm.

Fig 12 presents pressure waveforms measured at different anatomical sites for eight different aortas representing various aortic compliances (see Table 2 for their characteristics). Measured data corresponds to HR = 75 bpm and CO = 5 L/min. The raw data corresponding to all figures of this section is available online.

### 3.4. Effects of cardiac preload on coupled atrioventricular-aortic hemodynamics

**Fig 13:**
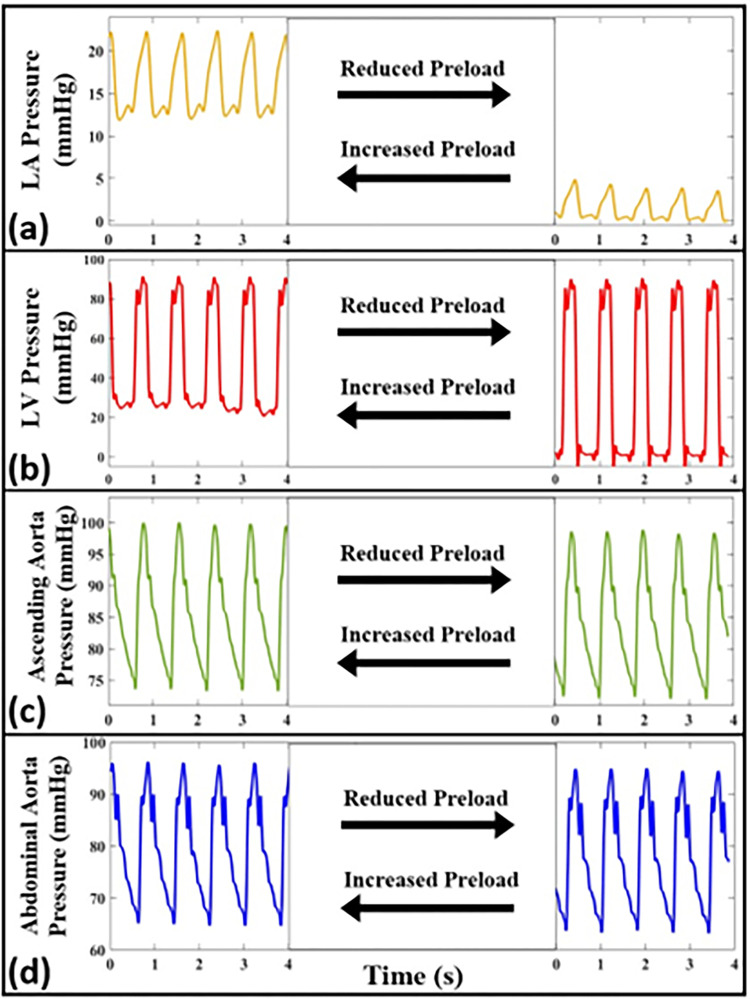
The measured pressure waveforms for increased and reduced preload conditions at different anatomical sites including **(a)** the left atrium, **(b)** the left ventricle, **(c)** the ascending aorta, and **(d)** the abdominal aorta. Waveforms are measured in Aorta-3 and correspond to HR = 75 bpm. The data of this figure is collected without pausing the pump to show the immediate dynamic changes of the setup (we only showed the oscillatory steady state of each phase for comparison).

Fig 13 demonstrates the effects of reduced and increased preload (relative to the baseline condition of Fig 5) on pressure waveforms measured at four different anatomical sites. The reduced and increased preload cases correspond to values of LVEDP corresponding to 1 mmHg and of 25 mmH*g*, respectively. The same heart rate (HR = 75 bpm), artificial aorta (Aorta-3), and contractility condition (normal) are considered for both measurements. The raw data corresponding to Fig 13 is available online.

### 3.5. Applicability of the setup for non-invasive measurements

**Fig 14:**
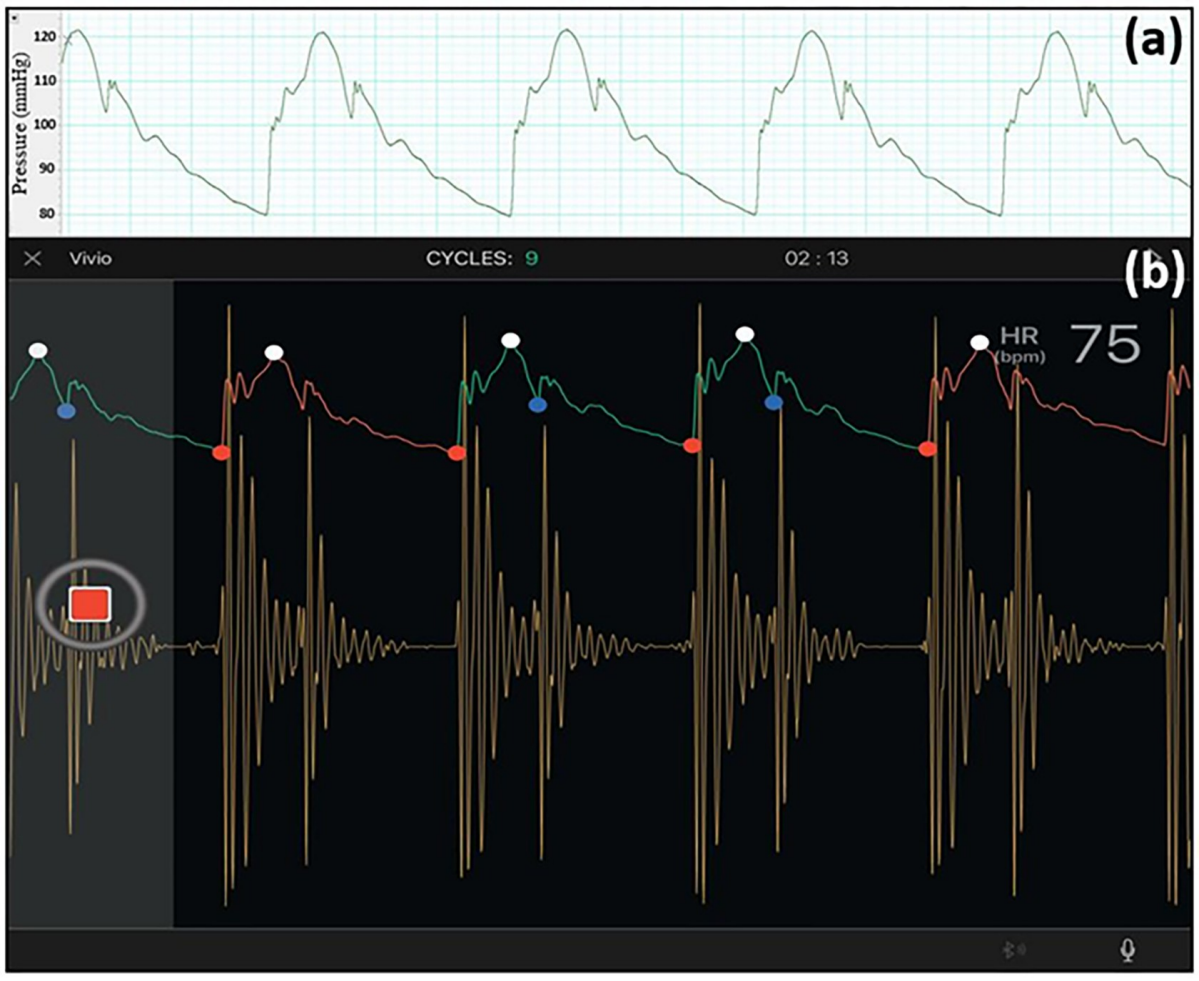
Example of simultaneous invasive and noninvasive measurements using **(a)** a Piezo-tip Millar MIKRO-TIP catheter transducer (each square accounts for 50 milliseconds), and **(b)** Vivio (a wireless optical tonometer [41]). Data is recorded for Aorta-4 at CO = 4.5 L/min and HR = 75 bpm. The Vivio system simultaneously measures pulse pressure and heart sound, communicating with a tablet or an iPad for real-time data display and for capturing via Bluetooth low energy (BLE).

Fig 14A and 14B present pressure waveform measurements using a high-fidelity Piezo-tip Millar pressure catheter (an invasive measurement) and an FDA-approved non-invasive handheld device (called Vivio [41]), respectively. Fig 14B shows a sample screenshot of the Vivio iPad application, illustrating the heart sound measured using Vivio. These measurements are conducted on Aorta-4 at CO = 4.5 L/min and HR = 75 bpm.

## 4. Discussion

In this paper, we have introduced and described a state-of-the-art experimental model for the in-vitro investigation of hemodynamics and wave propagation in the systemic circulation. Results demonstrate the ability of our in-vitro experimental setup to reproduce physiological functions of the LV, the LA, and the vascular system that correspond to both healthy and diseased conditions. These functions have been evaluated across a wide range of hemodynamic conditions (i.e., different afterload, preload, and contractility).

For assessing the physiological relevancy of the setup, we have measured the pressure waveforms at different anatomical sites of the system including inside the LV, inside the LA, the ascending aorta and the abdominal aorta (Fig 5). For the baseline pressure measurements, the LV-excitation pump has been operated at 75 bpm with a measured CO of 5 L/min (corresponding to normal contractility). As can be observed in Figs 5 and 6, our system is able to generate the main physiological features of the pressure and flow waveforms as found in the human cardiovascular system. These include: *i*) the physiological development of the pressure inside the LV and the aorta during systole (corresponding to the LV-arterial coupling), *ii*) the presence of the dicrotic notch due to the aortic valve closure (corresponding to the LV-arterial decoupling), *iii*) the interaction of the ventricle and the atrium during the filling phase of the LV via the mechanical mitral valve (corresponding to the LV-LA coupling and decoupling), *iv*) the increase in the pulse pressure as the wave propagates downstream towards the abdominal aorta, and *v*) the physiological point-to-point consistency of the pressure and flow [37–40]. Indeed, Figs 5 and 6 demonstrate that our setup generates physiologically-relevant waveform morphologies [37–40]. For the baseline hemodynamic measurement (Fig 5), the mean aortic pressure corresponds to 94.25 mmHg, the aortic SBP is 121.20 mmHg, the aortic DBP is 80.78 mmHg, and the total peripheral resistance (TPR) is 18.85 mmHg∙min/L. We also used two pulse wave analysis indices, augmentation index (AIx) and form factor (FF), for assessment of our waveform morphologies [40]. AIx represents an indication of the incidence of the reflected wave and is defined as $AIx=(\frac{AP}{cPP})\times 100$, which is the percentage of the augmented pressure (AP = cSBP-Pi) relative to the central pulse pressure (cPP) [44], where cSBP is the central systolic blood pressure (the maximum blood pressure value in the systole), and Pi is the blood pressure at inflection point. FF is a metric to quantify pulse waveforms and is defined as the ratio between the mean pulse pressure (MPP) and cPP. In the above definition, cPP = cSBP-DBP is the systo-diastolic change in arterial pressure [45], and MPP = MAP-DBP where MAP refers to the mean arterial pressure [40]. After computing AIx and *FF* based on the above definitions, the values of *AI* = 29.8% and *FF* = 0.33 were obtained, which are within the physiological ranges [40,44]. In order to evaluate the dynamic accuracy of the setup, we have also considered WI (Fig 7A and 7B), which is an energy-based clinical index quantifying the power carried in the arterial system (Section 2.4). The three major peaks of WI are successfully generated by our in-vitro setup for different cardiac outputs and aortas. We have shown in Fig 7A that as CO decreases, so does the amplitude of all three major peaks as expected. We have also employed another measure, aortic input impedance [37,46], for similarly assessing the dynamic accuracy of our system. As demonstrated in Fig 7C and 7D, our measured impedances fall well-within the expected physiological ranges and profiles [17,37].

We have further studied the effects of cardiac function on the measurements of our coupled in-vitro system. At a fixed cardiac output and LV contractility, it is observed (Fig 10) that higher heart rates lead to lower pulse pressures inside the aorta and the atrium (a result expected from previous studies [4,10]). Previous studies have shown the impact of heart rate on the arterial wave dynamics and have established this impact on the pulsatility of the propagated waves [4,10]. Indeed, heart rate along with the aortic characteristics and reflection sites are considered to be the major determinants of wave dynamics in the arterial system. Our model is shown to be sensitive to capturing the heart rate effects on pulse pressure as demonstrated in Fig 10. Additionally, at a fixed CO and HR, we have investigated the impact of LV contractility alone, achieved by changes in the input profile of the LV-excitation pump, on the propagated pressure waveforms. LV contractility is one of the major determinants of LV function and contributes to the state of ventricular-arterial coupling. Recent clinical studies have emphasized its importance in the context of wave propagation inside the vasculature [44,47]. For instance, Fok et al. [44] have recently shown a novel mechanism whereby augmentation pressure is influenced predominantly by the contractility and relaxation dynamics of the myocardium (contractility). In our measurements, the values for ascending aorta pulse pressure were measured as 54, 59, and 64 mmHg for low, normal, and high contractility, respectively (Fig 9). Also, the values for abdominal aorta pulse pressure were measured as 62, 64, and 72 mmHg for low, normal, and high contractility, respectively. Such measurements (also shown in Fig 9) indicate that impaired contractility, independently of the stroke volume, results in lower pulse pressure in the ascending and abdominal aorta as well as changes to waveform morphology. These results are consistent with previous studies related to the isolated effect of cardiac contractility on cardiovascular hemodynamics [44,48]. Pagoulatou et al. [49] have recently demonstrated the impact of low and high contractility on arterial wave propagation at a fixed heart rate. Their results suggest that an increase in cardiac contractility, with no concomitant change in arterial properties, alters the shape of the pressure wave [49] (a similar effect can be captured by our simulator). We have also further quantified the effects of changing LV contractility on the ascending aorta pressure waveform by calculating the value of *dp/dt*_*Max*_ for such waveforms. The *dp/dt*_*Max*_ values of the ascending aorta have been determined as *dp/dt*_*Max*_ = 443.4 mmHg/s for high contractility, *dp/dt*_*Max*_ = 404.8 mmHg/s for normal contractility, and *dp/dt*_*Max*_ = 358.0 mmHg/s for low contractility. The calculated aortic *dp/dt*_*Max*_ values verify the proper LV-arterial coupling at which different LV contractility conditions physiologically affect the propagated wave toward the arterial system in our in-vitro setup. Moreover, from the data presented in Fig 9, the peak values for low, normal, and high contractility conditions are 121, 123, and 128 mmHg, respectively, showing minor increases in peak LV pressure with a contractility increase at a fixed cardiac output (i.e., CO = 5 L/min). In in-vivo settings, the increase in contractility is also accompanied by increase in the cardiac output, where the combination of both parameters can lead to a more significant increase in peak pressure.

Our in-vitro setup is also a valuable tool for simulating cardiovascular diseases related to impaired cardiac function. We have successfully simulated physiological systolic dysfunction (from mild to severe) for different levels of cardiac output (Fig 8). The hemodynamic results from the experimental setup demonstrate that reduced cardiac output leads to lower mean and pulse pressures inside the LV, the ascending aorta, and the abdominal aorta. For example, the ascending aorta pulse pressure values were measured as 25, 33, 36, and 41 mmHg for CO = 2, 3, 4, and 5 L/min, respectively. However, as can be seen in Fig 8, reduced cardiac output results in higher pressures inside the LA, as well as higher LVEDP values (LVEDP value elevated by 29 mmHg from 4 mmHg for CO = 5 L/min to 33 mmHg for CO = 2 L/min). This is in agreement with clinical observations suggesting that the occurrence of heart failure is highly correlated with elevated LVEDP [39,50].

Previous studies have demonstrated that aortic compliance is a contributor of the pulsatile load on the global cardiovascular system and that the peripheral resistance is a contributor to the steady portion [51]. In this manuscript, we have presented studies on both these afterloads using our in-vitro setup, and their effects on in-vitro hemodynamics have been investigated. As supported by Fig 12, our in-vitro setup is able to capture the effects of aortic compliance on the ventricular and arterial pulse pressures. As expected [4,10,38], lower aortic compliances (larger stiffnesses) lead to higher ventricular and arterial pressures. Fig 11 presents our in-vitro simulations for increased peripheral resistance where it can be seen that, in addition to the expected rise in the mean pressure in the ascending and abdominal aorta, our setup is also able to capture the increase in ventricular pressure that arises from increased peripheral resistance (a demonstration that our design faithfully considers direct LV-aortic coupling). Another effect of increasing the peripheral resistance in our setup is the drop in CO from 4 L/min to 3.3 L/min. This demonstrates successful direct hemodynamic coupling of the LV sac and the aorta. It also demonstrates that the general pumping behavior in our system acts exactly similarly to the pumping characteristics of the heart (which is neither flow nor pressure source [3,4]).

The effects of cardiac preload (a major contributor to the cardiovascular system) on the coupled atrioventricular-aortic hemodynamics has been presented in Fig 13. As expected, the LA pressure and the diastolic part of the LV pressure increase significantly after increasing the cardiac preload (LVEDP increases by 24 mmHg) [39,50]. This effect illustrates the coupling characteristics between the LA and LV in our setup. Such results point to the physiological relevancy of our in-vitro coupled atrioventricular-aortic simulator for studying LV-diastolic dysfunction.

Lastly, we have shown the applicability of our setup for non-invasive hemodynamic measurements. Non-invasive measurement methodologies are of particular interest due to currently-in-development wearable technologies for monitoring and diagnostic purposes. We have compared simultaneous invasive and non-invasive measurements using our setup, and have demonstrated (Fig 14) that the non-invasive waveforms measured by Vivio [41] successfully represent morphological and physiological features (e.g., the pressure augmentation point or the dicrotic notch) of the corresponding invasive waveforms. Additionally, as can be observed in Fig 14B, both the first as well as the second heart sounds are successfully generated by our constructed setup. Our non-invasive measurements are also physiological and demonstrate consistent phonocardiogram waveforms [52,53]. It is worth mentioning that such non-invasive measurements can be conducted via the setup for any experimental configuration no matter what vasculature characteristics or hemodynamic conditions are applied [41,54]. The non-invasive measurements were also to provide an example that any newly developed non-invasive measurement device can be tested using this setup. Vivio is only one such example.

Overall, our proposed setup has the potential utility of ultimately improving understanding of the cardiovascular system as well as the underlying hemodynamic mechanisms in different cardiovascular diseases with a focus on two-way aortic-atrioventricular couplings. Studies of cardiovascular complications such as heart valve diseases, aortic dissection/coarctation, and hemorrhagic shock are difficult to perform in humans and animals due to the high-risk and invasive nature of such procedures. Our setup can be a practical in-vitro tool for preliminary studies of such complications prior to expensive, complicated, and high-risk in-vivo trials. The setup can be also utilized for assessing the accuracy of newly-developed non-invasive methodologies as discussed earlier in this section. Another utility of our setup is for deep learning or machine learning studies on the cardiovascular system, which usually require large training databases [55] (due to the dynamic complexity of the cardiovascular system affected by many contributors). Having large databases is essential to ensure lower estimation variances and hence better prediction performance. The setup can be easily run many times over to systematically generate large in-vitro databases for such studies. Systematic databases can be generated by applying pre-known grids (e.g., uniformly distributed) of any size to the input parameters of the setup, running the setup for each input configuration, and waiting for the stationary condition of each simulation. Although automatic physiological structural remodeling is not possible in our setup, its corresponding responses can be incorporated offline through the inputs of our system. The effect of structural remodeling and regulatory adaptations in pathophysiological conditions can be implemented in our setup using the following strategies: 1) structural remodeling such as changes in mechanical properties and/or geometrical alterations (e.g., those related to various diseases) can be incorporated by fabricating artificial organs with different characteristics (e.g., dilated LV, stiffened aorta, aorta with aneurysm, or enlarged LA); 2) regulatory adaptations that impact hemodynamics (e.g., HR, LV contractility, peripheral resistance, arterial compliance, and LV filling pressure) can be incorporated by adjusting the settings of the pump, vascular components, or both. Examples of the ability of our system to adjust to such conditions include (but are not limited to) changing the piston pump’s motion profile (S1 Fig in S1 File), the frequency of the piston, the slope of the pump profile, resistances of the end-organ outlets (via the clamps), end-organ outlet compliances (via the syringes), the height of the venous reservoir tank, and the manufacturing of organs of different geometries and stiffnesses. Considering acute myocardial infarction (MI) as an example of an acute event, the corresponding hemodynamics can be easily modeled by the aforementioned operational changes in our in-vitro setup by increasing HR via the piston frequency, by decreasing contractility via the piston motion profile, and by increasing LV filling pressure via adjusting the height of the venous reservoir (note here that additional regulatory changes might also exist in clinical MI cases based on biological variability and specific types of the MI, but these are beyond the scope of the present manuscript). Similarly, considering dilated cardiomyopathy as an example of a chronic disease, the corresponding cardiovascular pathophysiology can be modeled in our system by fabricating a dilated LV with a thinner wall to incorporate the related structural remodeling and by decreasing LV contractility, together with increasing HR, to account for cardiovascular regulations (note here again that other physiological adaptations may also occur in different dilated cardiomyopathy patients and under different etiologies).

### 4.1. Limitations and future work

Previous studies have shown that the non-Newtonian behavior of blood impacts the hemodynamics of the cardiovascular system [56,57]. However, it is well-established that the fluid viscosity has negligible effects on hemodynamic waves [4,37].

The purpose of this study is to understand the isolated effects of major contributors on cardiovascular performance, which is inherently difficult to investigate in in-vivo settings. The inter-dependent effects of such changes may co-exist in certain physiological settings [49]. For example, changes in preload and afterload can trigger LV remodeling (due to, e.g., heart compensatory dynamics) and consequently affect LV contractility. Studying the inter-dependencies of such contributors is a limitation of our presented in-vitro system. These effects may include isolated changes in cardiac function (e.g., contractilities, and heart rates), afterload (e.g., peripheral resistance and aortic impedance), and cardiac preload (e.g., LVEDP).

In this work, the relaxation of the LV sac is controlled by a combination of both piston outward movement and the compliance effects; however, this process in the physiological setting consists of passive LV relaxation followed by passive and active LA contractions. The difference between our system’s relaxation process versus the physiological depressurization can affect the diastolic part of our pressure measurements (e.g., the pressure waveform decay may change). For future studies, the above-mentioned details regarding the physiological LV relaxation can be included by modifying the LV excitation chamber and the relaxation process. Additionally, in the present design, the LA contraction mostly relies on its compliance. For a more detailed study of atrial hemodynamics in the future, the physiological atrial kick can be modelled by an external excitation mechanism. Such mechanism can be coupled with the artificial LA using the ports provided on the LA chamber.

Other future works include developing a large in-vitro database using our setup for resolving issues related to usually small-sized in-vivo databases to facilitate specific hemodynamics-based big-data studies [58–62] (more details were provided in Section 4.). In addition, as mentioned in Section 4, the setup can be used to simulate more complicated diseases, e.g., myocardial infarction, hemorrhagic shock, or aortic coarctation/dissection, where several physiological responses need to be simulated (i.e., changing our setup based on the main pathophysiology/abnormalities of the corresponding disease condition) [32,63].

## 5. Conclusions

The design and fabrication of an in-vitro cardiovascular simulator are presented in this study. We have demonstrated the physiological relevancy of the setup by studying the expected physiological features in the measured hemodynamic waveforms. Our setup can be used for modeling the effects of major contributors of the cardiovascular system such as LV contractility, afterload, and cardiac preload. Studying such effects ultimately improves understanding of the underlying hemodynamic mechanisms of different cardiovascular diseases. Additionally, due to no media change between our LV and aorta, non-invasive sound and pulse measurements are also available via the setup; hence our system can be employed for assessing the accuracy of any newly-developed non-invasive methodology. Our proposed setup is also useful for cardiovascular big-data studies, potentially providing large in-vitro databases for use in deep learning or machine learning studies. Ultimately, this setup is a practical in-vitro tool for preliminary cardiovascular studies prior to expensive and high-risk in-vivo trials.

## Supporting information

S1 File Example of a physiological input waveform for the piston pump displacement. (DOCX)

S2 File The detailed CAD design of the constructed setup. (PDF)
