# Supplementary material for: A coupled atrioventricular-aortic setup for in-vitro hemodynamic study of the systemic circulation: Design, fabrication, and physiological relevancy
Source: PLoS One. 2022 Nov 4;17(11):e0267765. doi: 10.1371/journal.pone.0267765 (PMC9635706; doi:10.1371/journal.pone.0267765)
Supplement: S1 File — (DOCX) [file pone.0267765.s001.docx]

**Supplemental File-1**

**Title:**

**A coupled** **atrioventricular-aortic setup for in-vitro hemodynamic study of the systemic circulation: Design, Fabrication, and Physiological relevancy**

**Short Title: A Coupled Atrioventricular-Aortic Setup**

**Authors’ names and affiliations:**

**Rashid Alavi ^1,¶^, Arian Aghilinejad ^1,¶^, Heng Wei ^1^, Soha Niroumandi ^1^, Seth Wieman ^2^, Niema M Pahlevan ^1,3,*^**

**^1^**- Department of Aerospace and Mechanical Engineering, University of Southern California, Los Angeles, CA, United States

**^2^**- Dornsife/Viterbi Machine Shop, University of Southern California, Los Angeles, CA, United States

**^3^**- Division of Cardiovascular Medicine, Keck School of Medicine, University of Southern California, Los Angeles, CA, United States

^----------------------------------------------------------------------------------^

**^¶^** These authors contributed equally to this work.

^*^ Corresponding author

**Address for correspondence:**

Professor Niema M. Pahlevan,

1002 Childs Way, MCB 470, Los Angeles, CA 90089, USA.

Telephone number: +1 (213) 740-7182

Email address: [pahlevan@usc.edu](mailto:pahlevan@usc.edu)


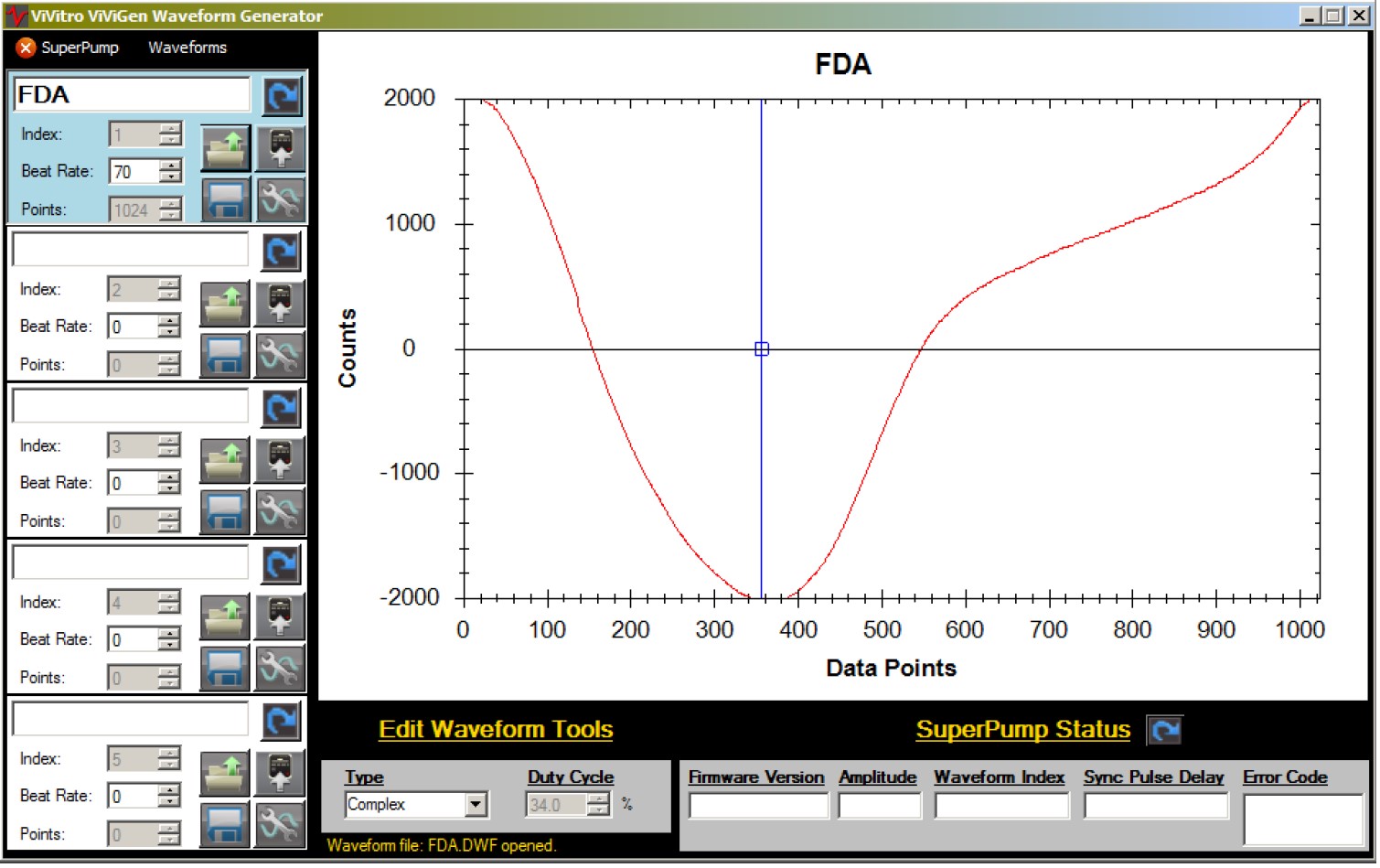


**Figure S1. Sample of an input waveform used for the pump displacement to achieve physiological heartbeats:** A predefined waveform (Physio-70) by the Vivitro SuperPump AR Series is used here to achieve the desired physiological heart rates.
