# Supplementary figures and images for: A coupled atrioventricular-aortic setup for in-vitro hemodynamic study of the systemic circulation: Design, fabrication, and physiological relevancy
Source: PLoS One. 2022 Nov 4;17(11):e0267765. doi: 10.1371/journal.pone.0267765 (PMC9635706; doi:10.1371/journal.pone.0267765)

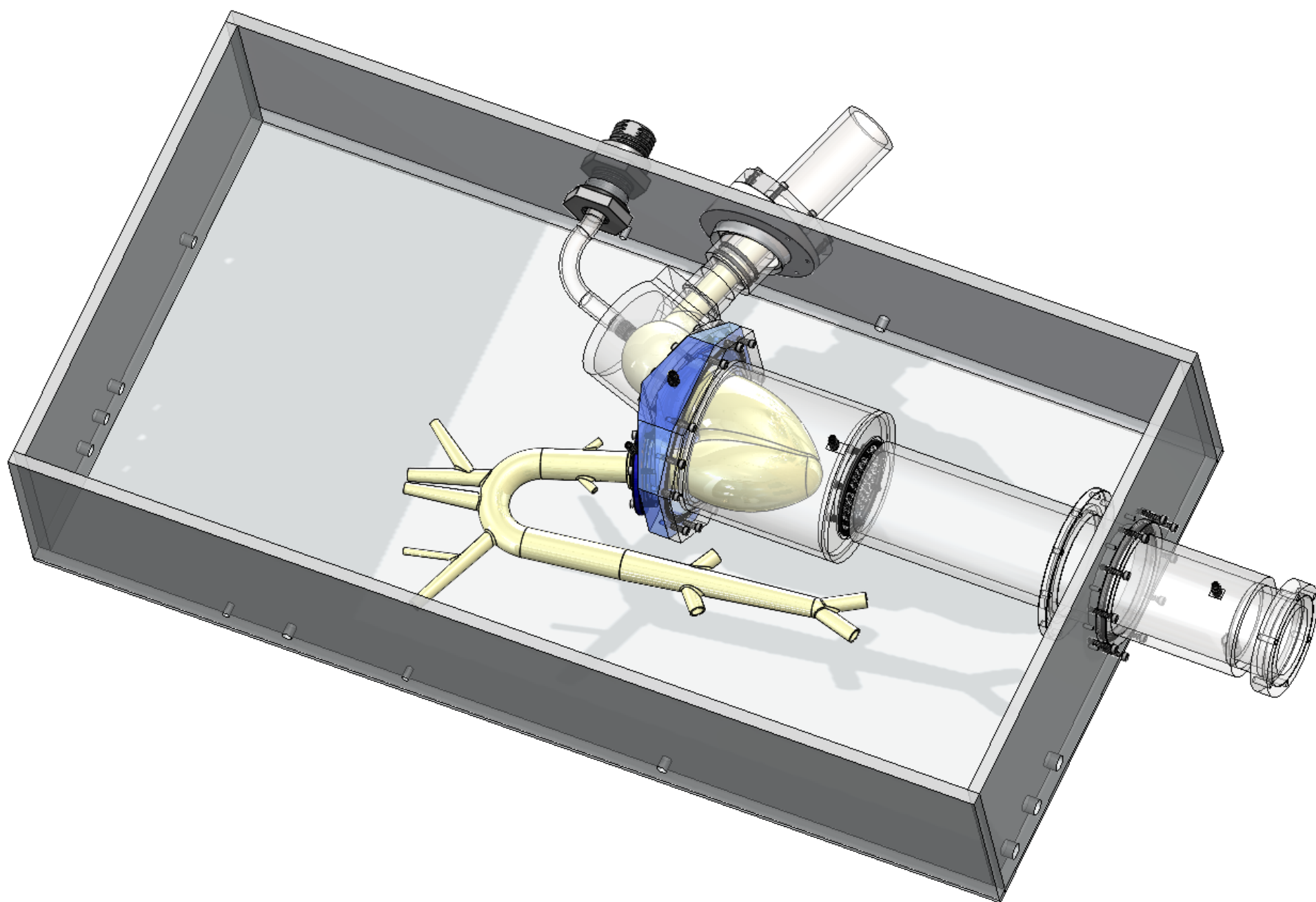

The 3D CAD files will be sent upon request. Please email [Alavideh@usc.edu](mailto:Alavideh@usc.edu)

Supplement: S2 File — (PDF) [file pone.0267765.s002.pdf]
